# Supplementary material for: The duration of antibiotic treatment is associated with carriage of toxigenic and non-toxigenic strains of Clostridioides difficile in dogs
Source: PLoS One. 2021 May 12;16(5):e0245949. doi: 10.1371/journal.pone.0245949 (PMC8115768; doi:10.1371/journal.pone.0245949)
Supplement: S1 File — (PDF) [file pone.0245949.s001.pdf]

S1 File Owner questionnaire for risk factors for *C. difficile* carriage in dogs and associations with clinical disease

Owner name____________________ Date___________

Small Animal Hospital ID_________________

Information on your dog

Name______________ Sex Male /Female (delete as necessary)

Breed_____________ Age ____________

Faecal sample provided? Yes ☐ No ☐ Normal faeces ☐ Diarrhoea ☐

1. What diet do you normally feed your dog?

   ____

   ____

   Please list any treats, titbits or food you occasionally feed your dog

   ____

   ____

2. Do you ever feed raw meat to your dog ?
   - ☐ Yes
   - ☐ No
3. Do you have any other pets in your home?
   - ☐ Yes
   - ☐ No

   Please tell us about the other pets in your home

   Total number of dogs____

   Total number of cats____

   Please list any other pets____

4. Can you tell us about the people who live in your home?

   Total number of people ?____

   Total number of infants (less than 2 years old)_________________________

   Total number of people over 65 years?_______________________________
