# Supplementary material for: The duration of antibiotic treatment is associated with carriage of toxigenic and non-toxigenic strains of Clostridioides difficile in dogs
Source: PLoS One. 2021 May 12;16(5):e0245949. doi: 10.1371/journal.pone.0245949 (PMC8115768; doi:10.1371/journal.pone.0245949)
Supplement: S1 Table — * F = Forward, R = Reverse. (PDF) [file pone.0245949.s002.pdf]

S1 Table. Primers and amplification conditions for 16S and (*tcdA* and *tcdB*) PCR

Target gene	F/R*	Primer sequence	PCR Conditions
16S ribosomal RNA	F<br><br>R	YT GGG YDT AAA GNG<br><br>CCC GTC AAT TYY TTT RAG TTT	95°C 5 min, 35 cycles 95°C, 50s, 50°C, 1min, 72°C, 1min; with a final extension 72°C, 10 mins.
*tcd*A	F<br><br>R	ATG TCT TTA ATA TCT AAA GAA GAG<br><br>CCT TTT TTA GTG TAT TGA CTA AG	95°C 2 min, 35 cycles 95°C, 30s, 52°C, 30s, 72°C 60s with a final extension of 72°C for 10 mins.
*tcd*B	F<br><br>R	ATG AGT TTA GTT AAT AGA AAA CAG<br><br>GTT TAT CAA AAA TGC ATT ACT ATC	95°C 5 min, 35 cycles 95°C, 50s; 50°C, 1 minute; 72°C, 60s with a final extension 72°C for 10 mins.

* F = Forward, R = Reverse
