# Supplementary material for: The duration of antibiotic treatment is associated with carriage of toxigenic and non-toxigenic strains of Clostridioides difficile in dogs
Source: PLoS One. 2021 May 12;16(5):e0245949. doi: 10.1371/journal.pone.0245949 (PMC8115768; doi:10.1371/journal.pone.0245949)
Supplement: S2 Table — *Based on comparative sequence of typical isolates of these ribotypes and the fragment amplified by PCR, this isolate would be expected to be positive by PCR. Strains of the ribotype 017 belong to the toxinotype VIII group, and are TcdA negative. However, as failure to produce TcdA is linked to a 1.8kb deletion at the 3’ end of the gene, amplification of the first 400bp should have been feasible and the result here is unexpected. (PDF) [file pone.0245949.s003.pdf]

S2 Table. *C. difficile* isolates from dogs in this study; ribotype, results of *tcd*A and *tcd*B PCR testing and cytotoxicity testing for active toxin presence.

Clinical symptoms	Ribotype	Isolates (n)	*tcd*A	*tcd*B	Active toxin detected/tested
Acute diarrhoea	009	2	0	0	0/1
	010	5	0	0	0/1
	026	1	1	1	0/1
	039	3	0	0	0/2
	078	1	1	1	NA
Chronic diarrhoea	010	2	0	0	0/1
	017*	1	0	1	NA
	039	6	0	0	NA
	039	1	1	1	NA
No diarrhoea	009	4	0	0	0/4
	010	6	0	0	0/2
	011	1	1	1	0/1
	012	1	1	1	0/1
	012	1	0	0	NA
	014	2	1	1	0/1
	020	9	1	1	1/4
	031	1	0	0	0/1
	039	8	0	0	0/4
	106	3	1	1	1/2
	154	1	1	1	0/1
	NA	1	0	0	NA
	NA	1	NA	NA	NA

*Based on comparative sequence of typical isolates of these ribotypes and the fragment amplified by PCR, this isolate would be expected to be positive by PCR. Strains of the ribotype 017 belong to the toxinotype VIII group, and are TcdA negative [1]. However, as failure to produce TcdA is linked to a 1.8kb deletion at the 3' end of the gene, amplification of the first 400bp should have been feasible and the result here is unexpected.

Reference

1. Rupnik M, Brazier JS, Duerden BI, Grabnar M, Stubbs SLJ. Comparison of toxinotyping and PCR ribotyping of *Clostridium difficile* strains and description of novel toxinotypes. Microbiology. 2001;147: 439–447. doi:10.1099/00221287-147-2-439
