# Supplementary material for: The duration of antibiotic treatment is associated with carriage of toxigenic and non-toxigenic strains of Clostridioides difficile in dogs
Source: PLoS One. 2021 May 12;16(5):e0245949. doi: 10.1371/journal.pone.0245949 (PMC8115768; doi:10.1371/journal.pone.0245949)
Supplement: S3 Table — Data from Health Protection Scotland Annual Reports 2015–2018 (1–3). (PDF) [file pone.0245949.s004.pdf]

S3 Table. Frequency of the most common ribotypes collected from human clinical surveillance for *Clostridium difficile* infection (CDI) in Scotland from mild, moderate or severe CDI cases (snapshot surveillance). Data from Health Protection Scotland Annual Reports 2015-2018 [1–3].

Ribotype	2015 Isolates (n)	%	2016 Isolates (n)	%	2017 Isolates (n)	%	2018 Isolates (n)	%
**001**	15	3.9	10	3.2	NA	NA	NA	NA
**002**	50	13.2	32	10.4	36	10.4	45	12.9
**005**	33	8.7	23	7.4	32	9.2	26	7.4
**012**	15	3.9	1	0.3	NA	NA	NA	NA
**014**	32	8.4	33	10.7	32	9.2	31	8.9
**015**	35	9.2	22	7.1	32	9.2	34	9.7
**020**	25	6.6	22	7.1	23	6.6	26	7.4
**023**	15	3.9	19	6.1	15	4.3	28	8.0
**026**	13	3.4	5	1.6	NA	NA	NA	NA
**027**	NA	NA	NA	NA	1	0.3	2	0.6
**078**	40	10.5	25	8.1	34	9.8	41	11.7
**106**	NA	NA	NA	NA	13	3.8	8	2.3
**Others**	107	28.2	117	37.9	128	37	108	30.9

References

1. Health Protection Scotland. Healthcare associated infections, Annual Report 2016. 2016. Available from: https://www.hps.scot.nhs.uk/web-resources-container/healthcare-associated-infection-annual-report-2016/. Accessed 7/10/2020
2. Health Protection Scotland. Healthcare Associated infection, Annual Report 2017. 2017. Available from: https://www.hps.scot.nhs.uk/publications/hps-weekly-report/volume-52/issue-18/hps-publish-2017-hai-annual-report/ Accessed 7/10/2020
3. Health Protection Scotland. Healthcare Associated infection Annual Report 2018. 2018. Available from: https://www.hps.scot.nhs.uk/web-resources-container/healthcare-associated-infection-annual-report-2018/. Accessed 7/10/2020
